# Association of Dual Decline in Cognition and Gait Speed With Risk of Dementia in Older Adults

**DOI:** 10.1001/jamanetworkopen.2022.14647

**Published:** 2022-05-31

**Authors:** Taya A. Collyer, Anne M. Murray, Robyn L. Woods, Elsdon Storey, Trevor T.-J. Chong, Joanne Ryan, Suzanne G. Orchard, Amy Brodtmann, Velandai K. Srikanth, Raj C. Shah, Michele L. Callisaya

**Affiliations:** 1 Peninsula Clinical School, Central Clinical School, Monash University, Frankston, Victoria, Australia; 2 Berman Center for Outcomes and Clinical Research, Hennepin Health Research Institute, Hennepin Healthcare and University of Minnesota, Minneapolis; 3 School of Public Health and Preventative Medicine, Monash University, Melbourne, Victoria, Australia; 4 Turner Institute for Brain and Mental Health, Monash University, Victoria, Australia; 5 Department of Neurology, Alfred Health, Melbourne, Victoria, Australia; 6 Department of Clinical Neurosciences, St Vincent’s Hospital, Melbourne, Victoria, Australia; 7 Florey Institute of Neuroscience and Mental Health, Heidelberg, Victoria, Australia; 8 University of Melbourne, Melbourne, Victoria, Australia; 9 Department of Family Medicine and Rush Alzheimer’s Disease Center, Rush University Medical Center, Chicago, Illinois

## Abstract

**Importance:**

Dual decline in gait speed and cognition has been found to be associated with increased dementia risk in previous studies. However, it is unclear if risks are conferred by a decline in domain-specific cognition and gait.

**Objective:**

To examine associations between dual decline in gait speed and cognition (ie, global, memory, processing speed, and verbal fluency) with risk of dementia.

**Design, Setting, and Participants:**

This cohort study used data from older adults in Australia and the US who participated in a randomized clinical trial testing low-dose aspirin between 2010 and 2017. Eligible participants in the original trial were aged 70 years or older, or 65 years or older for US participants identifying as African American or Hispanic. Data analysis was performed between October 2020 and November 2021.

**Exposures:**

Gait speed, measured at 0, 2, 4, and 6 years and trial close-out in 2017. Cognitive measures included Modified Mini-Mental State examination (3MS) for global cognition, Hopkins Verbal Learning Test-Revised (HVLT-R) for memory, Symbol Digit Modalities (SDMT) for processing speed, and Controlled Oral Word Association Test (COWAT-F) for verbal fluency, assessed at years 0, 1, 3, 5, and close-out. Participants were classified into 4 groups: dual decline in gait and cognition, gait decline only, cognitive decline only, and nondecliners. Cognitive decline was defined as membership of the lowest tertile of annual change. Gait decline was defined as a decline in gait speed of 0.05 m/s or greater per year across the study.

**Main Outcomes and Measures:**

Dementia (using *Diagnostic and Statistical Manual of Mental Disorders *[Fourth Edition] criteria) was adjudicated by an expert panel using cognitive tests, functional status, and clinical records. Cox proportional hazard models were used to estimate risk of dementia adjusting for covariates, with death as competing risk.

**Results:**

Of 19 114 randomized participants, 16 855 (88.2%) had longitudinal gait and cognitive data for inclusion in this study (mean [SD] age, 75.0 [4.4] years; 9435 women [56.0%], 7558 participants [44.8%] with 12 or more years of education). Compared with nondecliners, risk of dementia was highest in the gait plus HVLT-R decliners (hazard ratio [HR], 24.7; 95% CI, 16.3-37.3), followed by the gait plus 3MS (HR, 22.2; 95% CI, 15.0-32.9), gait plus COWAT-F (HR, 4.7; 95% CI, 3.5-6.3), and gait plus SDMT (HR, 4.3; 95% CI, 3.2-5.8) groups. Dual decliners had a higher risk of dementia than those with either gait or cognitive decline alone for 3MS and HVLT-R.

**Conclusions and Relevance:**

Of domains examined, the combination of decline in gait speed with memory had the strongest association with dementia risk. These findings support the inclusion of gait speed in dementia risk screening assessments.

## Key Points

**Question:**

Which cognitive measure among global cognition, memory, processing speed, and verbal fluency is most useful in assessing risk of future dementia when combined with gait decline?

**Findings:**

In this cohort study of 16 855 relatively healthy older people in Australia and the US, a dual decline in gait and cognitive function compared with nondecliners was significantly associated with increased risk of dementia. This risk was highest in those with both gait and memory decline.

**Meaning:**

These results highlight the importance of gait in dementia risk assessment and suggest that dual decline in gait speed and a memory measure may be the best combination to assess future dementia.

## Teaser

This cohort study using data from a large multinational clinical trial of older adults examines the association of dementia risk with a coinciding decline in gait and memory compared with domain-specific prognostic models.

## Introduction

The number of people with dementia is estimated to be 50 million worldwide and projected to grow to 150 million by 2050.^1^ As much of the neuropathology of dementia is believed to progressively accumulate 20 to 30 years before diagnosis,^2^ it is important that at-risk individuals are identified so that modifiable risk factors are addressed and available interventions provided.

Changes in motor performance are increasingly recognized as early markers of cognitive decline and dementia. Slow gait speed is associated with both cognitive decline and a greater risk of dementia.^3,4^ These associations may be because of underlying shared risk factors, such as cardiovascular disease, diabetes,^5^ and low physical activity,^6^ or common underlying neural pathways^7,8,9^ disrupted by cerebral small vessel disease^10^ or Alzheimer disease (AD) pathology.^11^ Over the past 10 years, studies have focused on improving sensitivity of motor biomarkers via combination with cognitive measures. For example, the presence of slow gait and subjective cognitive complaint (Motoric Cognitive Risk Syndrome [MCR]) has been associated with dementia over and above its individual components.^12^ This raises questions as to whether simultaneous (ie, dual) decline in gait and cognition over time is more strongly associated with future dementia risk than decline in either construct alone. Two previous studies showed stronger associations in those with dual decline compared with those showing no decline. However, these studies either had a small number of participants converting to dementia (22 participants),^13^ harmonized measures across diverse study conditions,^14^ and/or used limited cognitive measures (either global cognition^13^ or immediate memory^14^), and to our knowledge none have investigated nonamnestic cognitive domains. In most clinical settings it is unusual to apply a range of cognitive measures, especially domain-specific tests such as memory, attention, language, processing speed, and executive function. As it is already known that gait is more strongly correlated with executive function and processing speed,^15^ we hypothesized that, in addition to dual decliners having greater risk of dementia than nondecliners, the magnitude of elevated risk would be greatest in those with gait decline plus memory decline, as this measure would capture a broader range of cognitive domains and brain pathology. We aimed to examine these hypotheses using cognitive measures from a single, large clinical trial that assessed global cognition, processing speed, memory, and verbal fluency.

## Methods

### Study Population

Data were collected as part of the ASPREE (ASPirin in Reducing Events in the Elderly) trial (ClinicalTrials.gov identifier NCT01038583; International Standard Randomized Controlled Trial Number ISRCTN83772183). ASPREE was a double-masked, randomized, placebo-controlled trial conducted in Australia and the US of low-dose (100 mg) daily aspirin in 19 114 community-dwelling older people.^16^ Recruitment spanned 2010 to 2014 and randomized treatment concluded in 2017.^17^ Inclusion criteria were age 70 years or older (or ages 65 and older for US participants belonging to a minority group), free of cardiovascular disease, dementia, or physical disability (severe difficulty with 1 or more of Katz’s activities of daily living^18^) and expected to live longer than 5 years. During trial recruitment participants self-identified as Hispanic and/or 1 or more of the following: Aboriginal or Torres Strait Islander; American Indian; Asian; Black or African American; more than 1 race; Native Hawaiian, other Pacific Islander, or Maori; non-Hispanic White; and other. Absence of dementia at baseline was confirmed in writing by participants’ general practitioners and by cognitive screen (score of 78 or higher on the Modified Mini-Mental State examination [3MS]). All participants provided written informed consent, and ethics approval was granted by the ethics committees for Monash University and the Royal Australian College of General Practitioners in Australia and all participating clinic sites in the US.

### Exposures

Gait speed (in m/s) was measured at face-to-face visits at years 0, 2, 4, and 6 and the close-out visit in 2017. Participants completed 2 walks of 3 m at usual pace from standing start, with at least 1 meter at the end of the course to prevent slowing. The mean average of 2 walks was used for analysis.

Cognitive measures included a test of global cognitive function (3MS),^19^ delayed free recall (Hopkins Verbal Learning Test-Revised [HVLT-R-delay]),^20^ processing speed (Symbol Digit Modalities [SDMT]),^21^ and verbal fluency (Controlled Oral Word Association Test–single-letter version [COWAT-F]).^22^ Each was assessed at years 0, 1, 3, 5, and 2017 close-out.

### Outcomes

#### Dementia

Suspected cognitive concerns (3MS score below 78 or 10.15 points below predicted score; report of memory concerns to specialist, clinician diagnosis of dementia, prescription of cholinesterase inhibitors [Australia only]) triggered additional cognitive and functional assessment administered following a minimum 6-week delay to exclude delirium. These included the Alzheimer Disease Assessment Scale—Cognitive subscale, Lurian overlapping figures, and the Alzheimer Disease Cooperative Study Activities of Daily Living Scale.^23^ These data, plus available laboratory tests, brain scans, hospital, and/or specialist clinical case notes were considered by an international dementia end point adjudication committee comprising a panel of neurologists, neuropsychologists, and geriatricians from Australia and the US. The expert committee adjudicated dementia according to *Diagnostic and Statistical Manual of Mental Disorders* (Fourth Edition) (*DSM-IV*) criteria.^23^ Gait data were not used for end point adjudication.

### Statistical Analyses

Annual change in cognition and gait across the study period (prior to diagnosis of dementia, death, or last follow-up) was estimated for participants with longitudinal gait and cognitive data using multilevel linear regression, including random slopes and intercepts.^24^ Participants missing all data for 1 or more cognitive test were included in analysis of other tests, and participants missing data at particular time points were analyzed using other available time points. Gait decliners were classified using a cut-point of decline in gait speed of 0.05 m/s or more per year.^25^ Cognitive decliners were those in the lowest tertile of annual change in 3MS, HVLT-R-delay, SDMT,^14^ or COWAT-F scores. Participants were then classified into 4 phenotypic groups for each cognitive measure (3MS, HVLT-R-delay, SDMT, COWAT-F). For example, for 3MS, the 4 resultant groups were: (1) nondecliners, or those with less than 0.05 m/s annual decline in gait speed and in the highest two-thirds of annual change in 3MS; (2) cognitive decliners, who had less than 0.05 m/s annual decline in gait speed and in the lowest third (at greatest decline) of annual change in 3MS; (3) gait decliners, who had greater than 0.05 m/s annual decline in gait speed (at greatest decline) and in the highest two-thirds of annual change in 3MS; and (4) dual decliners, who had greater than 0.05 m/s annual decline in gait speed and who were also in the lowest third of annual change in 3MS.

We then employed Cox proportional hazards regression to examine whether group membership was associated with incident dementia. Death was modeled as a competing risk (via cause-specific hazard modeling^26^) and tied failures handled via the Breslow method. Resultant cause-specific hazards reflect the ratio of instantaneous risk of dementia, given participants were both alive and had not reached the dementia end point. Nondecliners formed the reference. As Kaplan-Meier survival estimates are inappropriate in the presence of competing risks,^26^ we present cumulative incidence curves. We adjusted for demographic characteristics (age, sex, education, and country) but did not adjust for comorbidities, as gait speed is included as an overall marker of the impact of these comorbidities on function. We did not statistically adjust for race or ethnicity in our models as numbers for these groups were low, and we did not hypothesize a unique association of gait and cognitive decline with subsequent dementia for racial or ethnic groupings. Randomization group was not included, as aspirin did not reduce cognitive decline, risk of dementia,^23^ or other primary end points.^27^

To evaluate whether changes in gait speed and each cognitive variable added prognostic value beyond baseline measurements, we compared 2 baseline-only models (baseline scores and a combined indicator of lowest cognitive and gait tertile membership) to longitudinal models using likelihood ratio tests. To determine if dual decliners had greater risk of dementia than gait or cognitive decliners, we performed linear contrasts of the relevant coefficients. α = .05 for all tests and statistical analyses were performed using Stata version 16 (Stata Corp).

## Results

**Figure 1.**
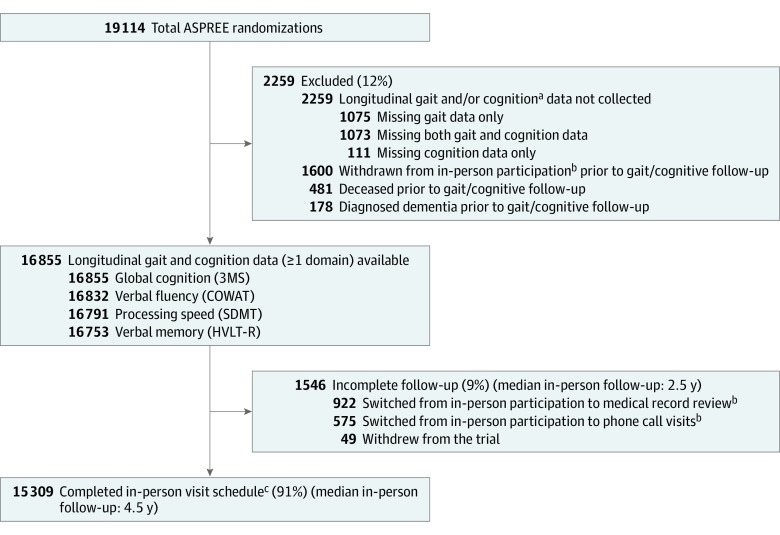
Study Flow Diagram 3MS indicates Modified Mini-Mental State examination; ASPREE, Aspirin in Reducing Events in the Elderly; COWAT, Controlled Oral Word Association Test; HVLT-R, Hopkins Verbal Learning Test-revised; SDMT, symbol digit modalities. ^a^Gait and cognitive data are missing in various combinations because of cognitive follow-up commencing at 12 months and gait follow-up commencing at 24 months. ^b^Dementia and death end point ascertainment continued for these participants until 2017, but cognitive testing and gait speed measures were not conducted via these follow-up modes. ^c^Until study end (2017) or death.

A total of 16 855 ASPREE participants (88.2%, of 19 114 total randomizations) had longitudinal gait and cognitive data available for analysis (Figure 1). Mean (SD) age was 75.0 (4.4) years, 9435 participants (56.0%) were women and 7558 (44.8%) reported education levels of 12 years or more (Tables 1 and 2). Across the 4 cognitive measures, 2259 participants were excluded from at least 1 model because of the absence of gait and/or cognitive follow-up data prior to dementia diagnosis, death, or withdrawal. Of these, 178 were diagnosed with dementia, 481 died, and 1600 withdrew from in-person study visits prior to collection of relevant follow-up data.

**Table 1.** Participant Characteristics for Global Cognition and Memory Tests

Characteristics	Participants, No. (%) (N = 16 855)					
	Nondecliners	Cognitive only	Gait only	Dual decliners	Total included	Excluded^a^
**Global cognition (3MS)**						
Total included participants	8436 (50.1)	4008 (23.8)	2842 (16.9)	1569 (9.3)	16 855 (100)	2259
3MS administrations, median (IQR)^b^	4 (3-4)	4 (3-4)	3 (3-4)	3 (3-4)	3 (3-4)	1 (1-2)
Age, mean (SD), y	74.4 (4.0)	75.9 (4.8)	74.8 (4.3)	76.6 (5.1)	75.0 (4.4)	76.0 (5.3)
Sex						
Men	3502 (41.5)	2029 (50.6)	1122 (39.5)	767 (48.9)	7420 (44.0)	912 (40.4)
Women	4934 (58.5)	1979 (49.4)	1720 (60.5)	802 (51.1)	9435 (56.0)	1347 (59.6)
Education ≥12 y	3498 (41.5)	2119 (52.9)	1119 (39.4)	822 (52.4)	7558 (44.8)	1078 (47.7)
Baseline 3MS score, mean (SD)	94.5 (4)	92.1 (5)	94.4 (4)	91.5 (5)	93.7 (4.5)	91.7 (5.2)
Baseline gait, mean (SD), m/s	1.00 (0.20)	0.93 (0.21)	1.17 (0.23)	1.09 (0.24)	1.02 (0.25)	0.94 (0.25)
Hypertension^c^	6084 (72.1)	3067 (76.5)	2113 (74.3)	1203 (76.7)	12 468 (74.0)	1727 (76.4)
Diabetes^d^	764 (9.1)	501 (12.5)	278 (9.8)	193 (12.3)	1737 (10.3)	308 (13.6)
Current/former smoker	3621 (42.9)	1866 (46.6)	1217 (42.8)	709 (45.2)	7413 (44.0)	1121 (49.6)
BMI	28.0 (4.6)	28.2 (4.7)	28.2 (4.7)	28.0 (4.7)	28.1 (4.7)	28.1 (5.0)
Polypharmacy^e^	1998 (23.7)	1114 (27.8)	757 (26.6)	490 (31.2)	4359 (25.9)	729 (32.3)
Race or ethnic group						
Black	273 (3.2)	207 (5.2)	123 (4.3)	75 (4.8)	678 (4.0)	223 (9.9)
Hispanic	174 (2.1)	114 (2.8)	64 (2.3)	37 (2.4)	389 (2.3)	99 (4.4)
White	7878 (93.4)	3624 (90.4)	2613 (91.9)	1431 (91.2)	15 546 (92.2)	1904 (84.3)
Other^f^	111 (1.3)	63 (1.6)	42 (1.5)	26 (1.7)	242 (1.4)	33 (1.5)
US participants	980 (11.6)	459 (11.5)	363 (12.8)	154 (9.8)	1957 (11.6)	454 (20.1)
Dementia end point	25 (0.3)	158 (3.9)	27 (1.0)	178 (11.3)	397 (2.4)	178 (7.9)
**Memory (HVLT-R)**						
Total included participants	8369 (50.0)	4007 (23.9)	2787 (16.6)	1590 (9.5)	16 753 (100)	2361
HVLT-R administrations, median (IQR)^b^	4 (3-4)	3 (3-4)	3 (3-4)	3 (3-4)	3 (3-4)	1 (1-2)
Age, mean (SD), y	74.3 (3.9)	75.9 (4.8)	74.7 (4.2)	76.7 (5.1)	75.0 (4.4)	76 (5.3)
Sex						
Men	3412 (40.8)	2089 (52.1)	1097 (39.4)	781 (49.1)	7379 (44.0)	953 (40.4)
Women	4957 (59.2)	1920 (47.9)	1691 (60.7)	809 (50.9)	9374 (56.0)	1408 (59.6)
Education ≥12 y	3502 (41.8)	2087 (52.1)	1128 (40.5)	799 (50.3)	7516 (44.9)	1120 (47.4)
Baseline 3MS score, mean (SD)	94.7 (5)	91.7 (5)	94.5 (4)	91.3 (5)	93.7 (4.5)	92.0 (5.1)
Baseline gait, mean (SD), m/s	1.00 (0.20)	0.94 (0.21)	1.17 (0.23)	1.09 (0.23)	1.02 (0.22)	0.94 (0.24)
Baseline HVLT-R, mean (SD)	8.6 (3)	6.6 (3)	8.5 (3)	6.2 (3)	7.8 (3)	6.8 (3)
Hypertension^c^	6085 (72.7)	3018 (75.3)	2062 (74.0)	1228 (77.2)	12 394 (74.0)	1801 (76.3)
Diabetes^d^	748 (8.9)	510 (12.7)	280 (10.0)	188 (11.8)	1727 (10.3)	318 (13.5)
Current/former smoker	3583 (42.8)	1879 (46.9)	1227 (44)	682 (43)	7371 (44)	1163 (49)
BMI	28.1 (4.6)	28.0 (4.7)	28.2 (4.7)	28.0 (4.7)	28.1 (4.7)	28.1 (5.0)
Polypharmacy^e^	1965 (23.5)	1125 (28.1)	749 (26.9)	488 (30.7)	4327 (25.8)	761 (32.2)
Race or ethnic group						
Black	267 (3.2)	213 (5.3)	121 (4.3)	76 (4.8)	677 (4.0)	224 (9.5)
Hispanic	206 (2.5)	82 (2.0)	59 (2.1)	42 (2.6)	389 (2.3)	99 (4.2)
White	7779 (93.0)	3658 (91.3)	2560 (91.9)	1452 (91.3)	15 449 (92.2)	2001 (84.8)
Other^f^	117 (1.4)	54 (1.3)	47 (1.7)	20 (1.3)	238 (1.4)	37 (1.6)
US participants	1002 (12.0)	434 (10.8)	320 (11.5)	194 (12.2)	1951 (11.6)	460 (19.5)
Dementia end point	27 (0.3)	152 (3.8)	25 (0.9)	130 (8.2)	388 (2.3)	187 (7.9)

Abbreviations: BMI, body mass index (calculated as weight in kilograms divided by height in meters squared); HVLT-R, Hopkins Verbal Learning Test-revised; 3MS, Modified Mini-Mental State examination.

^a^ Individuals without relevant cognitive and/or gait follow-up prior to dementia diagnosis (178 participants), death (481 participants), or withdrawal from in-person follow-up (1600 participants).

^b^ Administrations prior to withdrawal from in-person follow-up or dementia diagnosis.

^c^ Hypertension was defined as having systolic blood pressure >139 mm Hg or diastolic blood pressure >89 mm Hg, or undergoing pharmaceutical treatment for high blood pressure.

^d^ Self-report of diabetes or fasting blood glucose ≥126 mg/dL or on pharmaceutical treatment for diabetes.

^e^ Concurrent use of 5 or more medications at baseline.

^f^ Any category with fewer than 200 participants. This includes Aboriginal or Torres-Straight Island, American Indian, Asian, Native Hawaiian and other Pacific Islander and Maori, and those who indicated they were not Hispanic but did not indicate a race or ethnic group.

**Table 2.** Participant Characteristics for Processing Speed and Verbal Fluency Tests

Characteristics	Participants, No. (%) (N = 16 855)					
	Nondecliners	Cognitive only	Gait only	Dual decliners	Total included	Excluded^a^
**Processing speed (SDMT)**						
Total included participants	8510 (50.7)	3894 (23.2)	2931 (17.5)	1456 (8.7)	16 791 (100)	2323
SDMT administrations, median (IQR)^b^	3 (3-4)	4 (3-4)	3 (2-4)	3 (3-4)	3 (3-4)	1 (1-2)
Age, mean (SD), y	74.5 (4.6)	75.6 (4.5)	75.0 (4.5)	76.1 (4.8)	75.0 (4.4)	76.0 (5.4)
Sex						
Men	3715 (43.7)	1799 (46.2)	1250 (42.6)	626 (43.0)	7390 (44.0)	942 (40.6)
Women	4795 (56.3)	2095 (53.8)	1681 (57.4)	830 (57.0)	9401 (56.0)	1381 (59.4)
Education ≥12 y	3724 (43.8)	1872 (48.1)	1281 (4.4)	648 (44.5)	7525 (44.8)	1111 (47.8)
Hypertension^c^	6158 (72.4)	2963 (76.1)	2178 (74.3)	1117 (76.7)	12 417 (74.0)	1778 (76.5)
Baseline 3MS score, mean (SD)	94.0 (4)	93.3 (5)	93.4 (5)	93.3 (5)	93.7 (4.5)	92.0 (5.2)
Baseline gait, mean (SD), m/s	0.99 (0.20)	0.96 (0.20)	1.15 (0.23)	1.12 (0.25)	1.02 (0.22)	0.94 (0.25)
Baseline SDMT score, mean (SD)	37 (10)	38 (10)	36 (10)	38 (10)	37 (10)	33 (11)
Current/former smoker	3733 (43.9)	1737 (44.6)	1278 (43.6)	635 (43.6)	7383 (44.0)	1151 (49.5)
BMI	28.1 (4.6)	28.0 (4.7)	28.2 (4.7)	28.1 (4.8)	28.1 (5.0)	28.1 (4.7)
Polypharmacy^d^	2073 (24.4)	1023 (26.3)	783 (26.7)	453 (31.1)	4332 (25.8)	756 (32.5)
Race or ethnic group						
Black	315 (3.7)	163 (4.2)	137 (4.7)	59 (4.1)	674 (4.0)	227 (9.8)
Hispanic	205 (2.4)	83 (2.1)	71 (2.4)	30 (2.1)	389 (2.3)	99 (4.3)
White	7871 (92.5)	3593 (92.3)	2670 (91.1)	1352 (92.9)	15 486 (92.2)	1964 (84.5)
Other^e^	119 (1.4)	55 (1.4)	53 (1.8)	15 (1.0)	242 (1.4)	33 (1.4)
US participants	965 (11.3)	470 (12.1)	336 (11.5)	178 (12.2)	1950 (11.6)	461 (19.8)
Diabetes^f^	819 (9.6)	444 (11.4)	207 (7.1)	162 (11.1)	1733 (10.3)	312 (13.4)
Dementia end point	101 (1.2)	81 (2.1)	90 (3.1)	64 (4.4)	391 (2.3)	184 (7.9)
**Verbal fluency (COWAT-F)**						
Total included participants	8448 (50.2)	3985 (23.7)	3002 (17.8)	1397 (8.3)	16 832 (100)	2282
COWAT administrations, median (IQR)^b^	3 (3-4)	4 (3-4)	3 (3-4)	3 (3-4)	3 (3-4)	1 (1-2)
Age, mean (SD), y	74.7 (4.3)	75.1 (4.4)	75.3 (4.6)	75.7 (4.8)	75.7 (4.8)	76.0 (5.3)
Sex						
Men	3526 (41.7)	2000 (50.2)	1216 (40.5)	670 (48.0)	7412 (44.0)	920 (40.3)
Women	4858 (57.5)	1985 (49.8)	1786 (59.5)	727 (52.0)	9420 (56.0)	1362 (59.7)
Education ≥12 y	3506 (41.5)	2107 (52.9)	1205 (40.1)	730 (52.3)	7548 (44.8)	1088 (47.7)
Baseline 3MS score, mean (SD)	94.4 (4.1)	92.4 (4.8)	94.0 (4.3)	92.1 (4.9)	93.6 (4.5)	92.0 (5.1)
Baseline gait, mean (SD), m/s	0.99 (0.20)	0.95 (0.20)	1.14 (0.24)	1.12 (0.24)	1.02 (0.22)	0.94 (0.25)
Baseline COWAT-F score, mean (SD)	12.9 (4.6)	10.8 (4.3)	12.8 (4.5)	10.5 (4.1)	12.1 (4.6)	11.4 (4.6)
Hypertension^c^	6161 (72.9)	2980 (74.8)	2238 (74.6)	1069 (76.5)	12 448 (74.0)	1747 (76.6)
Diabetes^f^	811 (9.6)	454 (11.4)	299 (10.0)	173 (12.4)	1737 (10.3)	308 (13.5)
Current/former smoker	3692 (43.7)	1790 (44.9)	1321 (44.0)	600 (42.9)	7403 (44.0)	1131 (49.6)
BMI	28.0 (4.6)	28.4 (4.7)	28.1 (4.7)	28.2 (4.7)	28.1 (4.7)	28.1 (5.0)
Polypharmacy^d^	2045 (24.2)	1063 (26.7)	814 (27.1)	427 (30.6)	4349 (25.8)	739 (32.4)
Race or ethnic group						
Black	7881 (93.3)	3610 (90.6)	2775 (92.4)	1257 (90.0)	15 523 (92.2)	1927 (84.4)
Hispanic	176 (2.1)	112 (2.8)	63 (2.1)	38 (2.7)	389 (2.3)	99 (4.3)
White	275 (3.3)	205 (5.1)	116 (3.9)	82 (5.9)	678 (4.0)	223 (9.8)
Other^e^	116 (1.4)	58 (1.5)	48 (1.6)	20 (1.4)	242 (1.4)	33 (1.4)
US participants	913 (10.8)	526 (13.2)	336 (11.2)	182 (13.0)	1957 (11.6)	454 (19.9)
Dementia end point	103 (1.2)	79 (2.0)	98 (3.3)	60 (4.3)	340 (2.0)	63 (2.8)

Abbreviations: BMI, body mass index (calculated as weight in kilograms divided by height in meters squared); COWAT-F, Controlled Oral Word Association Test (single letter); SDMT, Symbol Digit Modalities.

^a^ Individuals without relevant cognitive and/or gait follow-up prior to dementia diagnosis (178 individuals), death (481 individuals), or withdrawal from in-person follow-up (1600 individuals).

^b^ Administrations prior to withdrawal from in-person follow-up or dementia diagnosis.

^c^ Hypertension was defined as having systolic blood pressure >139 mm Hg or diastolic blood pressure >89 mm Hg, or undergoing pharmaceutical treatment for high blood pressure.

^d^ Concurrent use of 5 or more medications at baseline.

^e^ Any category with fewer than 200 participants. This includes Aboriginal or Torres-Straight Island, American Indian, Asian, Native Hawaiian and other Pacific Islander or Maori, and those who indicated they were not Hispanic but did not indicate a race or ethnic group.

^f^ Self-report of diabetes or fasting blood glucose ≥126 mg/dL or on pharmaceutical treatment for diabetes.

Investigating multiple cognitive domains would be unnecessary if decline in 1 domain implied decline in other domains. There were varying degrees of overlap between the 4 different cognitive decliner groups (3MS, HVLT-R, SDMT, COWAT-F) and the 4 groups of dual decliners (gait-3MS, gait-HVLT-R, gait-SDMT, gait-COWAT-F) (eTables 1 and 2 in the Supplement). Overlap was not near-complete for any cognitive decline grouping, with the degree of overlap ranging from 38% (between HVLT-R and SDMT) to 59% (3MS and HVLT-R). The degree of overlap between dual-decline groupings similarly ranged from 38% (HVLT-R and SDMT) to 58% (3MS and HVLT-R).

### Associations Between Dual Decline and Risk of Dementia

#### Global Cognition

Gait-3MS dual decliners and gait-only and cognitive-only decliners had significantly higher dementia incidence rates than nondecliners after adjustment for demographic characteristics, baseline 3MS, and gait speed (Table 3). The hazard ratio (HR) was highest for those in the dual decline group, indicating a more than 20-fold increase in risk (HR, 22.2; 95% CI, 15.0-32.9). Upon linear contrast of HRs, dual decliners had a 5-fold greater risk of dementia compared with gait-only decliners (HR, 5.5; 95% CI, 3.8-8.1) and a 3-fold increased risk compared with cognitive-only decliners (HR, 3.1; 95% CI, 2.5-3.9).

**Table 3.** Associations for Each Decliner Group and Risk of Incident Dementia

Characteristic	Dementia rate/1000 PY (95% CI)	Demographics, adjusted cause-specific HR (95% CI)^a^	*P* value	Demographics plus baseline performance, adjusted cause-specific HR (95% CI)^b^	*P* value
Global cognition (3MS)					
No decline	0.6 (0.4-0.9)	1 [Reference]	NA	1 [Reference]	NA
Cognition only	8.2 (7.0-9.6)	9.7 (6.6-14.2)	<.001	7.1 (4.9-10.5)	<.001
Gait only	2.1 (1.5-3.1)	3.4 (2.1-5.5)	<.001	4.0 (2.5-6.6)	<.001
Dual decline	18.8 (15.8-22.3)	25.2 (17.5-37.8)	<.001	22.2 (15.0-32.9)	<.001
Memory (HVLT-R)					
No decline	0.68 (0.47-1.0)	1 [Reference]	NA	1 [Reference]	NA
Cognition only	8.0 (6.8-9.3)	11.1 (7.5-16.5)	<.001	7.6 (5.1-11.4)	<.001
Gait only	2.0 (1.4-3.0)	3.1 (1.8-5.3)	<.001	3.8 (2.3-6.5)	<.001
Dual decline	18.3 (15.4-21.8)	29.1 (19.5-43.5)	<.001	24.9 (16.5-37.6)	<.001
Processing speed (SDMT)					
No decline	2.6 (2.1-3.1)	1 [Reference]	NA	1 [Reference]	NA
Cognition only	4.1 (3.3-5.1)	1.5 (1.1-1.9)	.007	1.3 (1.0-1.7)	.06
Gait only	7.2 (5.9-8.9)	3.0 (2.3-4.0)	<.001	3.7 (2.8-5.0)	<.001
Dual decline	9.1 (7.1-11.6)	3.7 (2.8-4.9)	<.001	4.3 (3.2-5.8)	<.001
Verbal fluency (COWAT-F)					
No decline	2.8 (2.3-3.4)	1 [Reference]	NA	1 [Reference]	NA
Cognition only	4.7 (3.8-5.8)	1.4 (1.1-1.9)	.01	1.2 (0.9-1.6)	.14
Gait only	8.5 (7.0-10.3)	3.0 (2.3-4.0)	<.001	4.1 (3.1-5.4)	<.001
Dual decline	12.4 (9.9-15.4)	3.8 (2.9-5.1)	<.001	4.7 (3.5-6.3)	<.001

Abbreviations: 3MS, Modified Mini-Mental State examination; COWAT-F, Controlled Oral Word Association Test (single letter); HR, hazard ratio; HVLT-R, Hopkins Verbal Learning Test-Revised; PY, person-years; SDMT, Symbol Digit Modalities.

^a^ Adjusted for age at baseline, sex, years of education (<9, 9-11, 12, 13-15, 16, or 17-21 years) and country (Australia or US).

^b^ Additional adjustment for baseline gait speed and baseline cognitive scores.

#### Memory

**Figure 2.**
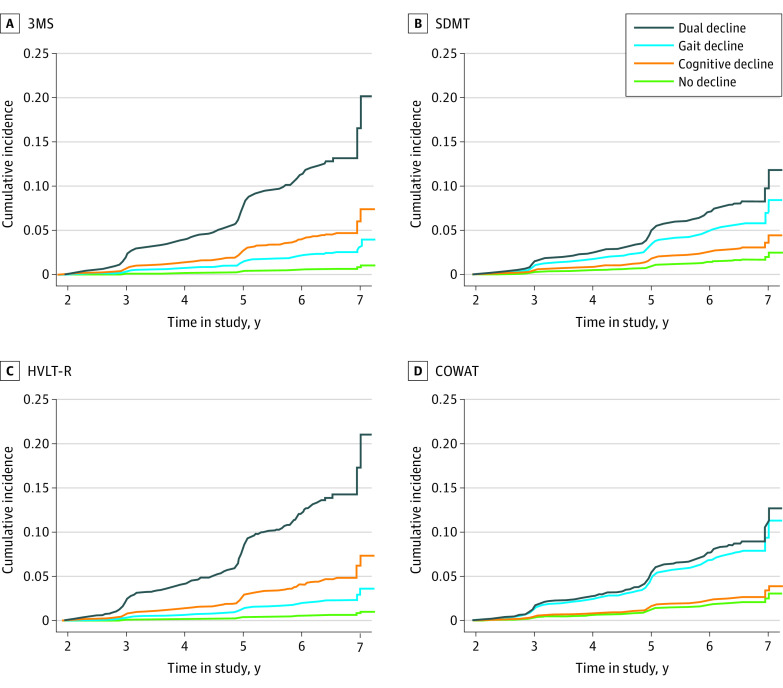
Cumulative Incidence of Dementia for Nondecliners, Gait Only, Cognition Only, and Dual Decliners 3MS indicates Modified Mini-Mental State examination; COWAT, Controlled Oral Word Association Test; HVLT-R, Hopkins Verbal Learning Test-revised; SDMT, symbol digit modalities.

Gait-HVLT-R-delay dual decliners and gait-only and cognitive-only decliners had a significantly higher risk of developing dementia compared with nondecliners (Table 3 and Figure 2). The highest HR was in the dual decline group (HR, 24.9; 95% CI, 16.3-37.3). Upon linear contrast review, dual decliners also had a greater risk of dementia compared with gait-only decliners (HR, 6.4; 95% CI, 4.2-9.8) and cognitive-only decliners (HR, 3.3; 95% CI, 2.6-4.1).

#### Processing Speed

Gait-SDMT dual decliners and gait-only decliners had a significantly higher risk of developing dementia compared with nondecliners (Table 3 and Figure 2). The highest hazard ratio (HR) was in those in the dual decline group (HR, 4.3; 95% CI, 3.2-5.8). On linear contrast, dual decliners had a greater risk of dementia compared with cognitive decliners (HR, 3.3; 95% CI, 2.4-4.4), but not gait-only decliners (HR, 1.2; 95% CI, 0.9-1.6).

#### Verbal Fluency

Gait-COWAT-F dual decliners and gait-only decliners had a significantly higher risk of developing dementia compared with nondecliners (Table 3 and Figure 2). The highest HR was for those in the dual decline group (HR, 4.7; 95% CI, 3.5-6.3). On linear contrast, dual decliners had a greater risk of dementia compared with the cognitive decliners (HR, 3.8; 95% CI, 2.8-5.2), but not gait-only decliners (HR, 1.1; 95% CI, 0.9-1.5).

### Comparison With Baseline

#### Continuous Measures

In Cox models (adjusted for age, education, gender, and country), higher baseline gait speed and cognitive test scores were both associated with lower dementia risk for all 4 models (eTable 4 in the Supplement). Compared with baseline-only models, models including baseline and longitudinal measures demonstrated a statistically significant improvement in fit on likelihood ratio tests (likelihood ratio χ^2^: 3MS, 337.73; *P* < .001; SDMT, 136.88; *P* < .001; HVLT-R-delay, 358.25; *P* < .001; COWAT, 152.31; *P* < .001) (Table 3).

#### Combined Cognition and Gait Measure

A binary indicator denoting simultaneous membership of the lowest cognitive and gait baseline tertiles was associated with higher dementia risk in all models (eTable 4 in the Supplement). Compared with these combined baseline indicator models, models including baseline and longitudinal measures demonstrated a statistically significant improvement in fit on likelihood ratio tests (likelihood ratio χ^2^: 3MS, 431.85; *P* < .001; SDMT, 131.95; *P* < .001; HVLT-R-delay, 457.30; *P* < .001; COWAT, 124.92; *P* < .001.).

## Discussion

This study examined associations between dual decline in gait speed and 4 different cognitive measures with incident diagnosis of dementia. The main findings from this study of over 16 000 older people were: (1) dual decline in gait speed and each of the cognitive measures was associated with higher risk of dementia when compared with nondecliners, cognitive only decliners, or gait only decliners (except for SDMT and COWAT-F measurements); (2) for dual decliners, risk of dementia was highest in the gait-HVLT-R-delay group, followed by the gait-3MS, gait-COWAT-F, and gait-SDMT groups; and (3) models that include these longitudinal decline groupings demonstrated superior goodness-of-fit to observed outcomes compared with models including baseline gait and cognitive scores only. These results highlight the importance of gait in dementia risk assessment and suggest that dual decline in gait speed and a memory measure may be the best combination associated with accurate assessment of future dementia risk.

Our findings expand on prior studies by testing 4 different cognitive measures in a single, large, well-characterized cohort. Importantly, we answer the question as to whether dual decline in gait speed and a test of processing speed or verbal fluency exhibits a similar association with progression to dementia as decline in memory (delayed recall) or global cognition. Descriptively, we found that the risk of dementia was highest in the memory dual decliners when compared with the global, verbal fluency, and processing speed dual decliners, which confirmed our hypothesis.

Prior studies have found slower gait speed in non-Alzheimer type dementia when compared with Alzheimer disease^28^; and others have demonstrated that gait speed is more strongly correlated with executive function and processing speed.^15^ Therefore, it is possible that gait measures capture decline in nonamnestic domains, which are required (in addition to memory decline) for dementia diagnosis using *DSM-IV* criteria applied in this study. Association between nonamnestic domains, such as processing speed and verbal fluency, with gait have been explained by the crossover in the underlying networks or pathology.^15,29,30,31,32^ In a prior study,^7^ gray matter covariance patterns consisting of the brain stem, precuneus, fusiform, motor, supplementary motor, and prefrontal cortex were associated with gait speed, processing speed, and executive function, but not memory. Furthermore, associations have been reported between cerebrovascular markers (white matter hyperintensities, microbleeds, and subcortical infarcts) and both poorer processing speed^29,30^ and slow gait speed.^32,33^ While global or overall memory performance shares overlapping networks with motor functions including gait, episodic memory also has distinct underlying networks reliant on normal hippocampal function.^34^ Poorer performance in tests of episodic memory is also more strongly linked to AD pathology, such as β-amyloid and tau accumulation.^35^ As the majority of cases of dementia are thought to be due to mixed pathology,^36^ the addition of gait speed to memory decline would necessarily capture a broader range of distributed brain networks.^37^

Our findings for global cognition are in agreement with a study of 135 older people with cognitive impairment (mean [SD] baseline Montreal Cognitive Assessment, 24.8 [3.5]), where dual decline in gait and cognition was associated with increased risk of dementia over 2 years compared with nondecliners.^13^ We expand on these findings with a larger cohort of healthy older people (mean [SD] baseline 3MS, 93.7 [4.5]). Our findings agree with those of Tian et al,^14^ who in a meta-analysis of 6 studies (8699 participants) found that dual decline in gait and a test of immediate verbal memory was associated with elevated dementia risk compared with usual agers. This meta-analysis harmonized several cognitive tests, criteria for dementia, and gait assessments over differing distances. Our study utilized consistent criteria and standardized tests for all participants. Importantly, we were able to test and confirm the hypothesis raised by Tian et al that memory may better differentiate phenotypic groupings, as decline in processing speed and verbal fluency are more strongly associated with decline in gait speed.^15,38^

The benefit of measuring both cognition and gait speed in studies of dementia risk has been previously established. A multicountry study found having MCR (a subjective cognitive complaint and poor gait speed) was associated with increased risk of dementia more than each of its components.^12^ We build on these findings in 2 ways. First, we showed that dual, longitudinal decline in cognition and gait improved modeling of incident dementia above baseline cognition and gait measures. Second, we demonstrated in a large sample that dual decline was associated with significantly higher risk of dementia than either gait-only or cognitive decline phenotypes (except when measured with SDMT and COWAT-F), suggesting that the combined measure has prognostic value. Measurement of gait speed has long been recommended in clinical practice as a marker of overall health and adverse outcomes such as falls, disability, hospitalization, and mortality.^39,40,41^ Our findings suggest that serial measurement of gait along with a simple test of memory would be more sensitive to future dementia risk than either measure alone. Such a test appears feasible in primary health clinics, although this requires confirmation in future implementation studies.

### Limitations

This study had several limitations. We reported secondary analysis of trial data; however, aspirin was not found to be associated with cognitive impairment or dementia^23^ and therefore was unlikely to have influenced our findings. Misclassification bias due to randomization of participants with dementia diagnosis cannot be fully excluded, but was unlikely because participants’ usual health care providers were closely involved during recruitment.^17^ Gait and cognition were not measured at the same time points, but our linear mixed-model approach accounted for this to some extent. However, a drawback of the mixed-model approach is that measurement error in longitudinal data and the correlation between random slopes and time to dementia diagnosis were unaccounted for in survival modeling.^42^

Censoring longitudinal data for mixed models at diagnosis date may have biased random slope estimates toward zero (ie, a less-steep decline) for those diagnosed with dementia. However, this would produce bias toward the null in survival models, and survival time and time-at-risk was not affected in our results.

The ASPREE sample is healthier than the general elderly population, and results may not generalize to less-healthy groups. As gait speed follow-up commenced 2 years after randomization, we could include only individuals who survived to year 2 and remained in the trial without dementia diagnosis. This means results were drawn from a healthier subgroup within ASPREE, potentially biasing baseline models, which did not include gait speed and cognitive test data for excluded participants.

Subtyping of dementia end points would have allowed exploration of the utility of dual decline in predicting dementia types but was unavailable. Dementia end point adjudications in ASPREE utilized the so-called “memory-plus” *DSM-IV* criteria, which may explain the stronger effect sizes observed for the associations with memory decline. Finally, it is to be expected that longitudinal decline in cognitive performance is strongly associated with dementia, as the former is a diagnostic criterion for the latter. By presenting specific comparisons between dual decline and cognitive decline groupings in this study, we have been able to specifically illustrate the additional benefit of a combined gait-cognition measure beyond cognitive testing alone.

## Conclusions

Dual decline in gait speed and cognition was associated with an increased risk of dementia in this study, with dual memory decliners showing greatest risk. Our findings provide further evidence for the importance of adding serial gait speed measures to dementia risk screening assessments, providing the opportunity for further comprehensive assessment and early preventative treatments.

## Notes

Supplement. **eTable 1.** Cognitive Change Within Tertiles **eTable 2.** Overlap Between Cognitive Decline and Dual Decline Groupings **eTable 3.** Participants Remaining At-Risk **eTable 4.** Baseline Cognitive Scores, Gait Speed and Association With Dementia in Baseline-Only Cox Models

## References

[1] Livingston G, Huntley J, Sommerlad A, . Dementia prevention, intervention, and care: 2020 report of the Lancet Commission. Lancet. 2020;396(10248):413-446. doi:10.1016/S0140-6736(20)30367-6 32738937 PMC7392084

[2] Jack CR Jr, Knopman DS, Jagust WJ, . Tracking pathophysiological processes in Alzheimer’s disease: an updated hypothetical model of dynamic biomarkers. Lancet Neurol. 2013;12(2):207-216. doi:10.1016/S1474-4422(12)70291-0 23332364 PMC3622225

[3] Jayakody O, Breslin M, Srikanth VK, Callisaya ML. Gait characteristics and cognitive decline: a longitudinal population-based study. J Alzheimers Dis. 2019;71(s1):S5-S14. doi:10.3233/JAD-181157 30958358

[4] Verghese J, Wang C, Lipton RB, Holtzer R, Xue X. Quantitative gait dysfunction and risk of cognitive decline and dementia. J Neurol Neurosurg Psychiatry. 2007;78(9):929-935. doi:10.1136/jnnp.2006.106914 17237140 PMC1995159

[5] Moran C, Beare R, Phan TG, Bruce DG, Callisaya ML, Srikanth V; Alzheimer’s Disease Neuroimaging Initiative (ADNI). Type 2 diabetes mellitus and biomarkers of neurodegeneration. Neurology. 2015;85(13):1123-1130. doi:10.1212/WNL.0000000000001982 26333802 PMC5573049

[6] Brach JS, Talkowski JB, Strotmeyer ES, Newman AB. Diabetes mellitus and gait dysfunction: possible explanatory factors. Phys Ther. 2008;88(11):1365-1374. doi:10.2522/ptj.20080016 18801861 PMC2579906

[7] Blumen HM, Brown LL, Habeck C, . Gray matter volume covariance patterns associated with gait speed in older adults: a multi-cohort MRI study. Brain Imaging Behav. 2019;13(2):446-460. doi:10.1007/s11682-018-9871-7 29629501 PMC6177326

[8] Jayakody O, Breslin M, Beare R, Srikanth VK, Blumen HM, Callisaya ML. The associations between grey matter volume covariance patterns and gait variability—the Tasmanian Study of Cognition and Gait. Brain Topogr. 2021;34(4):478-488. doi:10.1007/s10548-021-00841-5 33914190

[9] Rosano C, Studenski SA, Aizenstein HJ, Boudreau RM, Longstreth WT Jr, Newman AB. Slower gait, slower information processing and smaller prefrontal area in older adults. Age Ageing. 2012;41(1):58-64. doi:10.1093/ageing/afr113 21965414 PMC3234076

[10] Srikanth V, Beare R, Blizzard L, . Cerebral white matter lesions, gait, and the risk of incident falls: a prospective population-based study. Stroke. 2009;40(1):175-180. doi:10.1161/STROKEAHA.108.524355 18927448

[11] Del Campo N, Payoux P, Djilali A, ; MAPT/DSA Study Group. Relationship of regional brain β-amyloid to gait speed. Neurology. 2016;86(1):36-43. doi:10.1212/WNL.0000000000002235 26643548 PMC4731288

[12] Verghese J, Ayers E, Barzilai N, . Motoric cognitive risk syndrome: multicenter incidence study. Neurology. 2014;83(24):2278-2284. doi:10.1212/WNL.0000000000001084 25361778 PMC4277675

[13] Montero-Odasso M, Speechley M, Muir-Hunter SW, ; Canadian Gait and Cognition Network. Motor and cognitive trajectories before dementia: results from Gait and Brain Study. J Am Geriatr Soc. 2018;66(9):1676-1683. doi:10.1111/jgs.15341 29608780

[14] Tian Q, Resnick SM, Mielke MM, . Association of dual decline in memory and gait speed with risk for dementia among adults older than 60 years: a multicohort individual-level meta-analysis. JAMA Netw Open. 2020;3(2):e1921636. doi:10.1001/jamanetworkopen.2019.21636 32083691 PMC7043189

[15] Martin KL, Blizzard L, Wood AG, . Cognitive function, gait, and gait variability in older people: a population-based study. J Gerontol A Biol Sci Med Sci. 2013;68(6):726-732. doi:10.1093/gerona/gls224 23112113

[16] McNeil JJ, Woods RL, Nelson MR, ; ASPREE Investigator Group. Baseline characteristics of participants in the ASPREE (ASPirin in Reducing Events in the Elderly) Study. J Gerontol A Biol Sci Med Sci. 2017;72(11):1586-1593. doi:10.1093/gerona/glw342 28329340 PMC5861878

[17] Lockery JE, Collyer TA, Abhayaratna WP, . Recruiting general practice patients for large clinical trials: lessons from the Aspirin in Reducing Events in the Elderly (ASPREE) study. Med J Aust. 2019;210(4):168-173. doi:10.5694/mja2.12060 30835844 PMC6456041

[18] Katz S, Akpom CA. A measure of primary sociobiological functions. Int J Health Serv. 1976;6(3):493-508. doi:10.2190/UURL-2RYU-WRYD-EY3K 133997

[19] Teng EL, Chui HC. The Modified Mini-Mental State (3MS) examination. J Clin Psychiatry. 1987;48(8):314-318. 3611032

[20] Shapiro AM, Benedict RH, Schretlen D, Brandt J. Construct and concurrent validity of the Hopkins Verbal Learning Test-revised. Clin Neuropsychol. 1999;13(3):348-358. doi:10.1076/clin.13.3.348.1749 10726605

[21] Smith A. Symbol Digit Modalities Test. Western Psychological Services; 1973.

[22] Spreen O, Strauss E. A Compendium of Neuropsychological Tests. Administration, Norms, and Commentary. 2nd ed. Oxford University Press; 1998.

[23] Ryan J, Storey E, Murray AM, ; ASPREE Investigator Group. Randomized placebo-controlled trial of the effects of aspirin on dementia and cognitive decline. Neurology. 2020;95(3):e320-e331. doi:10.1212/WNL.0000000000009277 32213642 PMC7455352

[24] Diez-Roux AV. Multilevel analysis in public health research. Annu Rev Public Health. 2000;21:171-192. doi:10.1146/annurev.publhealth.21.1.171 10884951

[25] Perera S, Mody SH, Woodman RC, Studenski SA. Meaningful change and responsiveness in common physical performance measures in older adults. J Am Geriatr Soc. 2006;54(5):743-749. doi:10.1111/j.1532-5415.2006.00701.x 16696738

[26] Austin PC, Fine JP. Practical recommendations for reporting Fine-Gray model analyses for competing risk data. Stat Med. 2017;36(27):4391-4400. doi:10.1002/sim.7501 28913837 PMC5698744

[27] McNeil JJ, Wolfe R, Woods RL, ; ASPREE Investigator Group. Effect of aspirin on cardiovascular events and bleeding in the healthy elderly. N Engl J Med. 2018;379(16):1509-1518. doi:10.1056/NEJMoa1805819 30221597 PMC6289056

[28] Allali G, Annweiler C, Blumen HM, . Gait phenotype from mild cognitive impairment to moderate dementia: results from the GOOD initiative. Eur J Neurol. 2016;23(3):527-541. doi:10.1111/ene.12882 26662508 PMC4769662

[29] Debette S, Markus HS. The clinical importance of white matter hyperintensities on brain magnetic resonance imaging: systematic review and meta-analysis. BMJ. 2010;341:c3666. doi:10.1136/bmj.c3666 20660506 PMC2910261

[30] Prins ND, van Dijk EJ, den Heijer T, . Cerebral small-vessel disease and decline in information processing speed, executive function and memory. Brain. 2005;128(Pt 9):2034-2041. doi:10.1093/brain/awh553 15947059

[31] Srikanth V, Phan TG, Chen J, Beare R, Stapleton JM, Reutens DC. The location of white matter lesions and gait—a voxel-based study. Ann Neurol. 2010;67(2):265-269. doi:10.1002/ana.21826 20225293

[32] Choi P, Ren M, Phan TG, . Silent infarcts and cerebral microbleeds modify the associations of white matter lesions with gait and postural stability: population-based study. Stroke. 2012;43(6):1505-1510. doi:10.1161/STROKEAHA.111.647271 22442168

[33] Callisaya ML, Beare R, Phan TG, . Brain structural change and gait decline: a longitudinal population-based study. J Am Geriatr Soc. 2013;61(7):1074-1079. doi:10.1111/jgs.12331 23796055

[34] Jeong W, Chung CK, Kim JS. Episodic memory in aspects of large-scale brain networks. Front Hum Neurosci. 2015;9:454. doi:10.3389/fnhum.2015.00454 26321939 PMC4536379

[35] Maass A, Berron D, Harrison TM, . Alzheimer’s pathology targets distinct memory networks in the ageing brain. Brain. 2019;142(8):2492-2509. doi:10.1093/brain/awz154 31199481 PMC6658874

[36] Schneider JA, Arvanitakis Z, Leurgans SE, Bennett DA. The neuropathology of probable Alzheimer disease and mild cognitive impairment. Ann Neurol. 2009;66(2):200-208. doi:10.1002/ana.21706 19743450 PMC2812870

[37] Valenzuela MJ, Sachdev P. Brain reserve and dementia: a systematic review. Psychol Med. 2006;36(4):441-454. doi:10.1017/S0033291705006264 16207391

[38] Callisaya ML, Blizzard CL, Wood AG, Thrift AG, Wardill T, Srikanth VK. Longitudinal relationships between cognitive decline and gait slowing: the Tasmanian Study of Cognition and Gait. J Gerontol A Biol Sci Med Sci. 2015;70(10):1226-1232. doi:10.1093/gerona/glv066 26009641

[39] Montero-Odasso M, Schapira M, Soriano ER, . Gait velocity as a single predictor of adverse events in healthy seniors aged 75 years and older. J Gerontol A Biol Sci Med Sci. 2005;60(10):1304-1309. doi:10.1093/gerona/60.10.1304 16282564

[40] Studenski S, Perera S, Patel K, . Gait speed and survival in older adults. JAMA. 2011;305(1):50-58. doi:10.1001/jama.2010.1923 21205966 PMC3080184

[41] Studenski S, Perera S, Wallace D, . Physical performance measures in the clinical setting. J Am Geriatr Soc. 2003;51(3):314-322. doi:10.1046/j.1532-5415.2003.51104.x 12588574

[42] Albert PS, Shih JH. On estimating the relationship between longitudinal measurements and time-to-event data using a simple two-stage procedure. Biometrics. 2010;66(3):983-987. doi:10.1111/j.1541-0420.2009.01324_1.x 20849547

