# Error in Abstract

**DOI:** 10.1001/jamanetworkopen.2022.22274

**Published:** 2022-06-23

In the Original Investigation titled “Association of Dual Decline in Cognition and Gait Speed With Risk of Dementia in Older Adults,”^1^ published May 31, 2022, there was an error in a hazard ratio reported in the Abstract. The hazard ratio comparison for gait speed plus Hopkins Verbal Learning Test-Revised score decliners vs nondecliners should have appeared as 24.9 (95% CI, 16.5-37.6). This article has been corrected.^1^
