# Supplementary material for: Association of Dual Decline in Cognition and Gait Speed With Risk of Dementia in Older Adults
Source: JAMA Netw Open. 2022 May 31;5(5):e2214647. doi: 10.1001/jamanetworkopen.2022.14647 (PMC9157262; doi:10.1001/jamanetworkopen.2022.14647)
Supplement: Supplement. — eTable 1. Cognitive Change Within Tertiles eTable 2. Overlap Between Cognitive Decline and Dual Decline Groupings eTable 3. Participants Remaining At-Risk eTable 4. Baseline Cognitive Scores, Gait Speed and Association With Dementia in Baseline-Only Cox Models [file jamanetwopen-e2214647-s001.pdf]

Supplemental Online Content

Collyer TA, Murray AM, Woods RL, et al. Association of dual decline in cognition and gait speed with risk of dementia in older adults. *JAMA Netw Open.* 2022;5(5):e2214647. doi:10.1001/jamanetworkopen.2022.14647

eTable 1. Cognitive Change Within Tertiles

eTable 2. Overlap Between Cognitive Decline and Dual Decline Groupings

eTable 3. Participants Remaining At-Risk

eTable 4. Baseline Cognitive Scores, Gait Speed and Association With Dementia in Baseline-Only Cox Models

This supplemental material has been provided by the authors to give readers additional information about their work.

© 2022 Collyer TA et al. *JAMA Network Open.*

eTable 1. Cognitive Change Within Tertiles

Average estimated annual change in raw score			
	Lowest Tertile (95% CI)	Middle Tertile (95% CI)	Upper Tertile (95% CI)
3MS	-0.22 (-0.23, -0.22)	+0.04 (0.040, 0.041)	+0.20 (+0.193,0.198)
SDMT	-0.30 (-0.30, -0.29)	+0.01 (0.011, 0.012)	+0.28 (0.28, 0.29)
HVLT-R	-0.17 (-0.175, -0.168)	+0.026 (0.025, +0.027)	+0.16 (0.15, 0.16)
COWAT-F	-0.14 (-0.151, -0.148)	-0.009 (-0.010, -0.008)	+0.16 (0.15, 0.16)
Inter-Quartile Range of estimated annual change (25th percentile, 75th Percentile)			
	Lowest Tertile	Middle Tertile	Upper Tertile
3MS	-0.29, -0.09	+0.01, +0.08	+0.14, +0.23
SDMT	-0.38, -0.15	-0.02, +0.05	+0.16, +0.36
HVLT-R	-0.22, -0.09	-0.01, +0.06	+0.12, +0.19
COWAT-F	-0.19, -0.09	-0.04, +0.04	+0.07, +0.20

3MS - Modified Mini-Mental State examination; HVLT-R- Hopkins Verbal Learning Test-Revised; SDMT- Symbol Digit Modalities; COWAT-F - Controlled Oral Word Association Test (single letter)

© 2022 Collyer TA et al. *JAMA Network Open.*

eTable 2. Overlap Between Cognitive Decline and Dual Decline Groupings

A. Cognitive Decline Groupings					
	Total	Lower tertile HVLT-R	Lower tertile SDMT	Lower tertile 3MS	Lower tertile COWAT
Lower tertile HVLT-R	5,597 (100%)		2,140 (38%)	3,271 (58%)	2,421 (43%)
Lower tertile SDMT	5,350 (100%)	2,140 (40%)		2,197 (41%)	2,078 (39%)
Lower tertile 3MS	5,577 (100%)	3,271 (59%)	2,197 (39%)		2,433 (44%)
Lower tertile COWAT	5,382 (100%)	2,421 (44%)	2,078 (39%)	2,433 (45%)	
**B. Dual Decline Groupings**					
	Total	Dual Decline HVLT-R	Dual Decline SDMT	Dual Decline 3MS	Dual Decline COWAT
Dual Decline HVLT-R	1,590 (100%)		604 (38%)	907 (57%)	639 (40%)
Dual Decline SDMT	1,456 (100%)	604 (41%)		643 (44%)	558 (38%)
Dual Decline 3MS	1,569 (100%)	907 (58%)	643 (41%)		667 (43%)
Dual Decline COWAT	1,397 (100%)	639 (46%)	558 (40%)	667 (48%)	

HVLT-R- Hopkins Verbal Learning Test-Revised; SDMT- Symbol Digit Modalities; 3MS - Modified Mini-Mental State examination; COWAT-F - Controlled Oral Word Association Test (single letter).
Dual-Decline - Longitudinal decline in gait speed of ≥0.05m/s/y *plus* annualised cognitive change in lowest tertile of sample.

© 2022 Collyer TA et al. *JAMA Network Open.*

eTable 3. Participants Remaining At-Risk
(Cumulative Hazard Function for Dementia, Death as Competing Risk)

	Years Since Randomisation					
**Cognitive Measure**	2	3	4	5	6	7
**Global Cognition (3MS)**						
No Decline	8436	7473	5804	3666	1415	48
Cog Only	4008	3662	2908	1908	655	33
Gait Only	2842	2371	1647	990	342	6
Dual Decline	1567	1347	926	567	169	4
**Memory (HVLT-R)**						
No Decline	8369	7468	5839	3710	1453	53
Cog Only	4007	3622	2846	1851	606	30
Gait Only	2349	2349	1641	996	321	7
Dual Decline	1588	1348	923	557	187	4
**Attention-Speed of processing (SDMT)**						
No Decline	8510	7351	5599	3459	1251	33
Cog Only	3894	3756	3101	2106	808	48
Gait Only	2928	2335	1532	879	269	5
Dual Decline	1363	1363	1030	671	241	5
**Verbal fluency (COWAT-F)**						
No Decline	8447	7376	5630	3482	1295	39
Cog Only	3756	3756	3080	2091	767	42
Gait Only	3000	2429	1628	965	304	7
Dual Decline	1397	1281	940	590	204	4

3MS - Modified Mini-Mental State examination; HVLT-R- Hopkins Verbal Learning Test-Revised; SDMT-Symbol Digit Modalities; COWAT-F - Controlled Oral Word Association Test (single letter)

© 2022 Collyer TA et al. *JAMA Network Open.*

eTable 4. Baseline Cognitive Scores, Gait Speed and Association With Dementia in Baseline-Only Cox Models

	Baseline Score Model				Combined Baseline Indicator Model	
	Baseline Gait Speed		Baseline Cognitive Score		Indicator Variable: Lowest Tertile for cognition *and* Gait Speed at baseline	
	HR	95% CI	HR	95% CI	HR	95% CI
Global Cognition (3MS score)	0.56	0.35 - 0.90	0.85	0.84 - 0.87	3.20	2.57 – 4.00
Processing Speed (SDMT score)	0.60	0.38 - 0.97	0.93	0.92 - 0.94	2.93	2.33 - 3.67
Memory (HVLT-R score)	0.45	0.28 - 0.72	0.75	0.73 - 0.78	3.16	2.54 – 3.91
Verbal Fluency (COWAT-F score)	0.41	0.25 –0.66	0.94	0.91 – 0.96	1.86	1.48 - 2.36

All models also adjusted for age at randomisation, gender, education and country (Australia/US). HR = Hazard Ratio
3MS - Modified Mini-Mental State examination; HVLT-R- Hopkins Verbal Learning Test-Revised; SDMT-Symbol Digit Modalities; COWAT-F - Controlled Oral Word Association Test (single letter).

© 2022 Collyer TA et al. *JAMA Network Open.*
